# Binary Monolayers Formed from the Sequential Adsorption of Terphenylthiol and Dodecanethiol on Gold

**DOI:** 10.1021/acs.jpcc.5c00571

**Published:** 2025-05-19

**Authors:** Elizabeth Garrett, Sabrina Tang, Emma K. Canning, Daniel J. Williams, Aidan F. Bergin, Elaine Kelly, Luke Wadzinski, Emma R. Robinson, Alissandra Conlon, Jack Sette-Ducati, Sophia Renzi, Elizabeth C. Landis, L. Gaby Avila-Bront

**Affiliations:** Department of Chemistry, 8717 College of the Holy Cross, 1 College Street, Worcester, Massachusetts 01610, United States

## Abstract

Binary self-assembled monolayers (SAMs) of 1-dodecanethiol (DDT) and 1,1′,4′,1″-terphenyl-4-thiol (TPT) were prepared via sequential deposition on the surface of Au(111) on mica. The SAMs were studied using scanning tunneling microscopy (STM), X-ray photoelectron spectroscopy (XPS), cyclic voltammetry (CV), and reductive desorption (RD). Varying the sequence of deposition and the deposition temperature of DDT resulted in four distinct binary monolayer systems. Characterization of DDT SAMs with STM exhibited a combination of ordered and disordered domains, whereas the TPT monolayers were well-ordered into various phases and aligned with extensive characterization in the literature. Binary SAMs displayed varied behaviors, including retention of the initial SAM structure, adsorption of the secondary compound at domain boundaries, or temperature-dependent replacement of the initial SAM. Elemental analysis via XPS revealed the presence of oxygen in single-component DDT monolayers, which was absent in TPT monolayers, and the binary SAMs exhibited nearly identical elemental compositions. RD results indicate well-ordered domains for DDT, less ordered and less strongly bound TPT regions, and distinct domains for binary SAMs that were consistent with the binary STM results. CVs for single-component and binary SAMs of DDT and TPT indicate an absence of significant defects in the molecular layers. The inhibited electron transfer observed aligns with prior studies for DDT and is more moderate for TPT and mixed SAMs. This study addresses the need for an experimental understanding of the phase behavior of binary SAMs.

## Graphical abstract

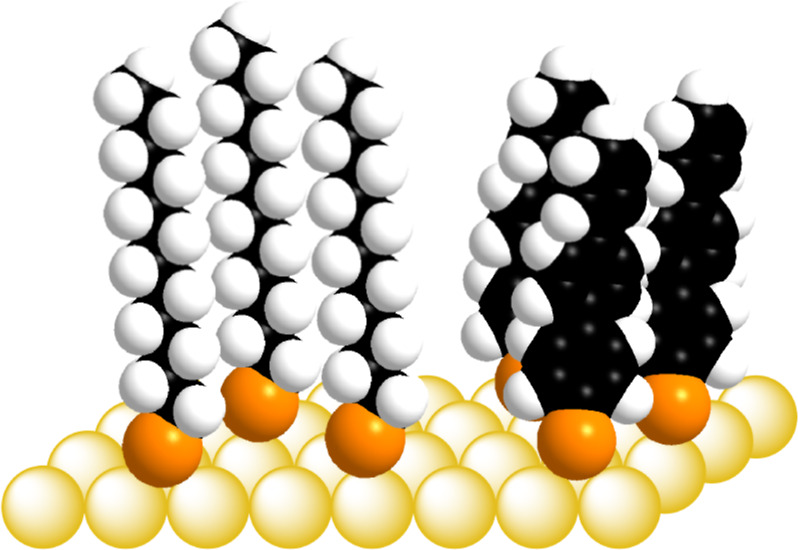

## Introduction

Self-assembled monolayers (SAMs) are highly ordered molecular films formed by the chemisorption and spontaneous assembly of functionalized molecules on surfaces.^1–11^ The adsorbed molecules relocate via surface diffusion, coalescing into islands (domains) that fill in the monolayer. As these domains come into contact with each other, fault lines (domain boundaries) are formed if the regions are not structurally aligned.^12,13^ As packing density increases, a phase transition occurs in which the molecules transition from this lying down phase to an upright, “crystalline phase” of ordered arrays.^14,15^ The molecular arrangement and properties of SAMs are strongly influenced by deposition conditions (e.g., immersion time, solvent, temperature) and molecular structure (e.g., chain length, conjugation, functional groups).^16–26^ SAMs of organothiols on coinage metals such as Au(111) are particularly well-studied due to the strong thiol–gold binding and utility in surface modification, molecular electronics, and nanoscale patterning.^27–30^ These applications, however, are heavily influenced by the surface structure of the SAM, and the ability of adsorbed molecules to form homogeneous surface structures.^31,32^ Therefore, understanding and controlling the domains and surface defects of organothiol self-assembled monolayers is extremely important in designing these electronic systems.

While single-component alkanethiol SAMs form relatively homogeneous and well-characterized monolayers, binary SAMs composed of two distinct molecules offer a promising route to engineer surfaces with tailored functionalities.^25,33–37^ Such systems can combine distinct molecular properties (e.g., conductivity, hydrophobicity) into one layer. However, achieving homogeneous mixing at the molecular level remains a major challenge due to differences in adsorption kinetics, intermolecular interactions, and steric compatibility, often leading to phase separation or kinetically trapped structures during assembly.^38–43^ Effective binary SAMs for nanotechnology applications have proven to be difficult to form due domain boundaries, phase separations, or defects that inhibit the conductivity of the surface.^44^

In particular, phase-separated domains can lead to local variations in electronic properties such as tunneling barriers, dielectric environment, and charge transport pathways.^1,45^ For applications where uniform molecular-level properties are desired, such as molecular electronics, sensing, or surface patterning, such heterogeneity could be detrimental, introducing variability or unpredictability in device performance.^45,46^ On the other hand, controlled phase separation and well-defined domain boundaries can be advantageous to, for example, enable spatial patterning of surface functionality or allow for engineered heterojunctions.^47,48^ Future work could address these issues by (1) tuning molecular design to balance size, shape, and interaction energies between components, (2) exploring alternative deposition protocols, such as sequential deposition, coadsorption, or using solvent annealing to promote rearrangement and mixing, (3) using surface patterning or templating strategies to guide the spatial arrangement of different components, and (4) combining experimental approaches with computational modeling to predict favorable mixing conditions and to design molecules that are more likely to assemble homogeneously. Ultimately, understanding and controlling the thermodynamic and kinetic factors governing assembly will be key to engineering homogeneous binary SAMs for targeted functional applications.

STM is widely used to characterize the surface topography and electronic reactivity of SAMs.^49–52^ This technique is helpful to determine the packing structures of adsorbed compounds as well as their orientation relative to the surface. Because of the highly local nature of STM measurements, electrochemical analysis is also used to analyze the stability, structure, degree of order, and the electrochemical environment of adsorbed SAMs. Reductive desorption is an electrochemical technique in which an increasingly negative potential is applied to the sample. The negative potential will cause adsorbed molecules to desorb from the surface following the equation^53–55^

$$RSAu+e^{-}\to {RS}^{-}+Au$$

The potential at which desorption occurs provides insight into the binding energy of the molecular layers.^56^ The shape of reduction peaks in cyclic voltammograms indicates how ordered the adsorbed molecules are. Wide, broad peaks indicate that the electrochemical environment of adsorbed molecules is more variable. This is understood to mean that there is a lack of order among adsorbed molecules.^57,58^ On the other hand, narrow peaks indicate that the electrochemical environment is fairly homogeneous across the SAM suggesting a more ordered SAM.^59^ Reductive desorption is useful when it comes to the analysis of alkanethiol monolayers because they have been shown to form highly ordered SAMs leading to sharp and defined peaks.^21,55,57,60,61^ Reductive desorption of mixed molecular layers has shown that the technique can be used to characterize phase separated domains and identify the absence of domain formation in binary SAMs.^62^ Controlled reductive desorption of phase separated SAMs has subsequently been used for surface structure formation, demonstrating the utility of domain separated SAMs for micro and nanoscale surface manipulation.^63,64^

Cyclic voltammetry can also be collected in the presence of a redox couple such as Fe­(CN)_6_^3–/4–^ to evaluate the coverage of the SAM.^65–67^ Bare gold electrodes will exhibit a diffusion limited redox process while dense SAM formation can block heterogeneous electron transfer at the surface.^1,68,69^ The shape of the cyclic voltammograms can therefore be used to determine the extent to which the SAM is free of pinholes and defects.

Characterizing the quality and structure of binary SAMs allows for the ability to apply this knowledge to the formation of new SAMs composed of related and relevant chemical species. In this work, binary SAMs formed by the sequential adsorption of DDT and TPT were studied on a Au(111)-on-mica surface. Deposition conditions of each of these species were performed under conditions to approach maximal coverage of the surface by the SAM and avoid submonolayer coverage. Varying conditions were investigated with the ultimate goal of understanding the surface structures of the compounds. TPT was chosen due to its highly conjugated structure, holding promise for forming a conductive monolayer.^70,71^ When paired with an insulating molecule,^72^ TPT can be used for the formation of functional SAMs, such as in the construction of nanowires.^73^ DDT was selected due to its formation of fairly homogeneous SAMs, as well as its similarity in length to TPT. This study is the first to characterize the SAM resulting from the sequential deposition of these two species, as well as the first reported RD measurements of TPT. Due to the broad applications of well-ordered, defect-less SAMs, this research is critical for technological advancements in the field of surface chemistry.

Previous studies from our group on binary SAMs involving alkanethiols and aromatic thiols, octanethiol/biphenyl-4-thiol,^38^ octanethiol/2-naphthalenethiol,^39^ and octanethiol/1-naphthalenethiol,^40^ reveal that the sequence and conditions of deposition significantly affect the resulting SAM structure. First, sequential adsorption often leads to phase separation, resulting in distinct domains characteristic of each thiol component. For example, in the octanethiol and biphenyl-4-thiol system, STM imaging showed separate domains with features specific to each compound, indicating limited mixing at the molecular level. In the 2-naphthalenethiol and octanethiol system, varying the deposition sequence led to different surface structures, with 2-naphthalenethiol being displaced by octanethiol when 2-naphthalenethiol had been deposited at a lower concentration. Next, oxidation has been found to impact SAMs of aromatic thiols inhomogeneously. Of the three systems described here, only 1-naphthalenethiol showed significant oxidation. The aromatic thiol’s susceptibility to oxidation influenced the monolayer’s structural characteristics and led to a highly disordered surface structure.

The study presented here offers valuable insights for designing functional SAMs for applications in which surface structure and molecular order are critical. STM and RD reveal that TPT, though less tightly bound, can form ordered domains, offering potential for conductive pathways. CV data confirm minimal defects across systems, supporting their use in insulating layers or tunneling junctions. XPS shows DDT is more prone to oxidation, highlighting considerations for material stability. Together, these findings provide a framework for engineering SAMs with targeted functionality through controlled deposition strategies.

## Methods

Samples were created using Au(111) on mica substrates (Phasis). These samples were flame annealed using a hydrogen flame. SAM samples were kept under a dark cover throughout deposition to avoid photooxidation.

### Deposition of Single-Component SAMs

DDT monolayers were prepared by immersing the bare, flame-annealed substrate in a scintillation vial into a 1.0 mM ethanolic solution of DDT (Sigma-Aldrich) for 1 h at two different temperatures–either 78 °C in a water bath (DDT(78)), or room temperature (DDT­(RT)). Samples were removed, rinsed with pure ethanol, and dried with nitrogen gas. A range of deposition temperatures and adsorption times were tested during method optimization. Room temperature was chosen as a baseline condition, as it represents a mild, commonly used environment for SAM formation.^74^ 78 °C was selected because it represents a moderate thermal treatment that is often used in SAM literature^75^ to promote better molecular packing and potentially encourage the formation of more ordered and/or larger domains. Adsorption times of 1, 2, 4, 18, 20, and 24 h were also tested for the deposition of DDT. However, from our observations, none of the longer deposition times consistently produced significantly better results. Because there was not a clear difference in monolayer quality, the shorter, 1 h deposition was ultimately chosen for ease of preparation.

TPT monolayers were prepared by immersing the bare, flame-annealed substrate in a scintillation vial into a 0.1 mM ethanolic solution of TPT (Sigma-Aldrich) for 2 h in a 50 °C oven (Quincy Lab, 20E-LT Lab Oven). Samples were removed, rinsed with ethanol, and dried with nitrogen gas.

### Deposition of Binary SAMs

We refer to binary SAMs in which DDT was adsorbed first and then immersed in TPT solutions as DDT/TPT. Similarly, SAMs in which existing TPT monolayers were exposed to DDT solutions are referred to as TPT/DDT. As in our notation above, we distinguish between DDT temperatures as either DDT(78) or DDT­(RT). Binary SAMs of TPT/DDT were prepared using TPT deposition conditions, including ethanol rinsing and nitrogen drying, followed by DDT deposition conditions. DDT/TPT SAMs were generated using DDT deposition conditions followed by rinsing and drying, followed by TPT deposition conditions.

### STM Parameters

Samples were scanned on STM (Bruker, Multimode 8 with Scan Assist) immediately after final ethanol rinsing and nitrogen drying. STM tunneling tips were manually cut from 0.25 mm diameter platinum iridium wire (80% Pt, 20% Ir) (Goodfellow Corp). The STM was stabilized with an air table (Technical Manufacturing Corporation) to eliminate scanning noise. Standard scanning parameters had an aspect ratio of 1.00, scan angle of 0°, and sample lines of 512. Images were scanned at a rate of 2.96 Hz. Standard feedback parameters had a current set point of 100.0 pA, integral and proportional gains of 1.00, and sample bias of 100.0 mV.

### X-ray Photoelectron Spectroscopy (XPS)

Elemental analysis was performed using a Thermo Scientific K-Alpha + X-ray Photoelectron Spectrometer with a monochromatic Al Kα X-ray source. Spectra were collected with a 50 eV pass energy, a 25 ms dwell time, and a 0.1 eV step size. The number of scans collected were 20 for C­(1s), 2 for Au­(4f), 30 for S­(2p), and 20 for O­(1s). Peaks were fit using Thermo Advantage software and relative areas were corrected using sensitivity factors. Samples were removed from their deposition vials and transferred to dark containers for transport 1–2 h before analysis.

### Electrochemistry

Electrochemistry measurements were performed by attaching the Au(111) samples described above to glass slides with epoxy for ease of handling. Electrical contact to the surface of the sample was achieved by attaching conductive copper tape to the gold surface. The copper tape was coated in nail polish so that only the gold surface was exposed to the electrolyte solution. A picture is included in Figure S1. Samples were placed in an electrochemical cell with a Basi Ag/AgCl reference electrode and a Pt wire counter electrode. Measurements were performed on a Pine Research Wave Driver 100 potentiostat. Reductive desorption was performed in 0.5 M KOH in milli-Q water from 0 V to −1.6 V with a scan rate of 0.1 V/s. Measurements were collected on at least four samples and uncertainties represent the standard deviation of the measured values. Cyclic voltammetry in ferricyanide solution was performed in 1.0 mM K_3_Fe­(CN)_6_ and 1.00 M KCl from −0.1 to 0.6 V at a scan rate of 0.1 V/s.

## Results and Discussion

Figure 1 shows the chemical structures of the compounds investigated in this study. Measured between the sulfur atom and the twelfth carbon atom, DDT has an approximate length of 15.441 Å. TPT has an approximate length of 13.427 Å from the outer edge of its farthest benzene ring to the sulfur atom. Both measurements were obtained using the software Avogadro.^76^

**Figure 1:**
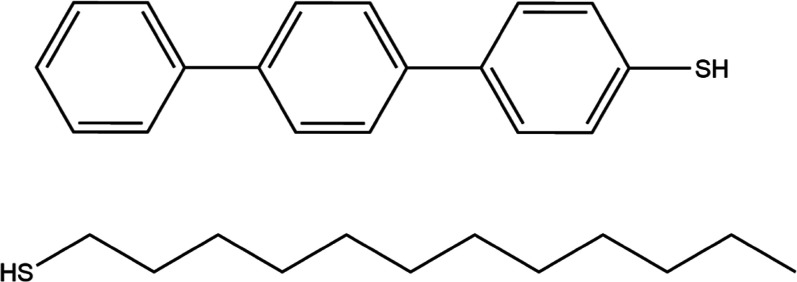
Chemical structures the compounds investigated in this study: 1,1′,4′,1″-terphenyl-4-thiol (TPT) and 1-dodecanethiol (DDT).

### STM Images of Single-Component Monolayers of DDT and TPT

Figures 2 and 3 show representative STM images of the single-component DDT and TPT monolayers, respectively. Both DDT^75,77^ and TPT^20,78–85^ SAM structures have been thoroughly characterized in the literature. STM imaging of DDT SAMs in the literature reveals small vacancy islands (etch pits), and distinct domains of standing-up and lying-down phases, as well as disordered phases. Studies of TPT SAMs via STM and complementary techniques reveal a mixture of highly complex surface structures impacted by immersion time and temperature, annealing times and temperatures, deposition phase (solution or vapor), deposition solvent, and electron exposure. Data revealed polymorphism with distinct structural phases, demonstrating the influence of imaging conditions and phase coexistence.

**Figure 2:**
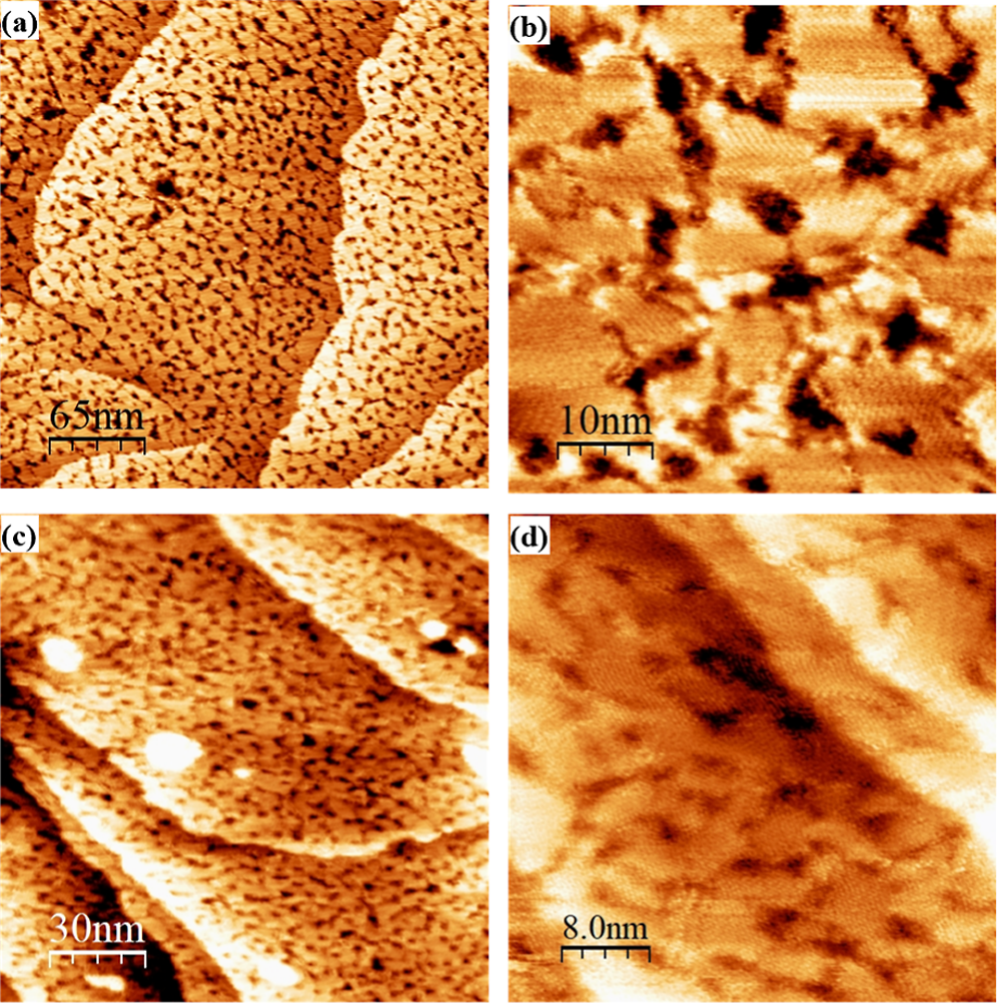
Representative STM images of DDT deposited on Au(111) for 1 h. Panels (a,b) show a representative STM image of samples deposited in a water bath held at 78 °C, and panels (c,d) show a representative STM image of samples deposited at room temperature. In all depositions, samples were immersed in 1 mM ethanolic solution, rinsed with ethanol, and dried under a nitrogen stream.

**Figure 3:**
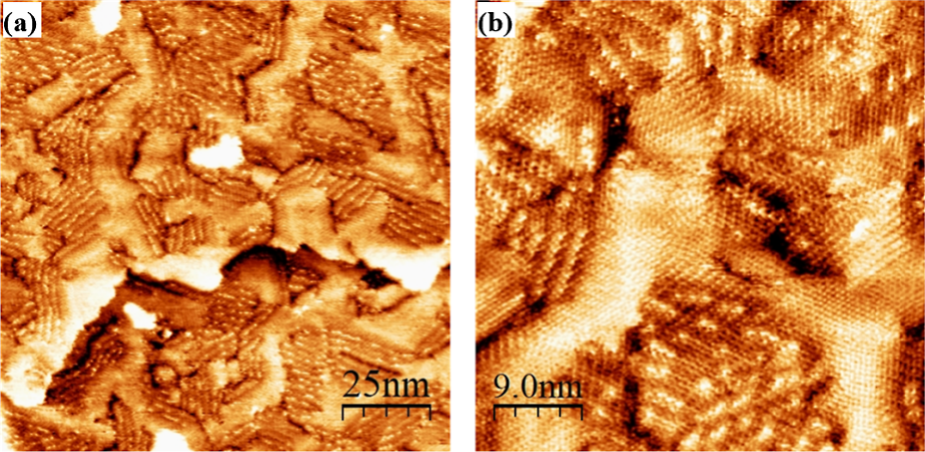
Representative STM image of single-component TPT formed by a 2 h immersion at 50 °C in a 0.1 mM ethanolic solution, rinsed with ethanol and dried in a nitrogen stream. TPT SAMs are marked by a myriad of two-dimensional phases in images (a,b).

Our own STM images are in agreement with previously published reports investigating DDT on the (111) surface of gold. We observed large flat areas marked by domain boundaries and etch pits with a high variability of size. Molecular resolution was not achieved on all samples. In regions where molecular resolution was achieved, we observed hexagonally packed molecules, and an average distance between molecules in the same row as 0.40 ± 0.05 nm and between molecules across different rows as 0.50 ± 0.04 nm. These measurements are consistent with the (√3 × √3)­R30° structure as well as previous reports in the literature.^77^

In general, TPT SAMs formed well-structured multiphase monolayers such that molecular resolution was easily achieved on many domains and samples. Our investigation is consistent with the literature in finding a mixture of structural phases. The structural phases can be distinguished from one another by differences in heights (and therefore false color contrast) and geometric differences.^83^ Packing density and nearest neighbor measurements depend on the phase and surface direction, as discussed in the literature.

### STM Images of Binary Monolayers of DDT/TPT and TPT/DDT

#### DDT/TPT

Binary monolayers of DDT and TPT were created via sequential adsorption, as aforementioned. When existing DDT monolayers were immersed in TPT solutions, the resulting surface structures were characterized either by features specific to single-component DDT monolayers, or ones in which disordered areas surrounded flat domains. Both types of structures were observed regardless of the deposition temperature of the DDT solution. Figure 4 shows representative images of the two observed surface structures. In panel (a), we observe a large terrace in between two other terraces in the upper left-hand and lower right-hand corners of the image. All of the terraces have etch pits scattered throughout. As etch pits are indicative of the adsorption of an alkanethiol monolayer, we conclude that these areas had DDT present or still have DDT molecules. Relying solely on STM images, we cannot definitively determine whether the DDT molecules remain on the surface because molecular resolution was not achieved.

**Figure 4:**
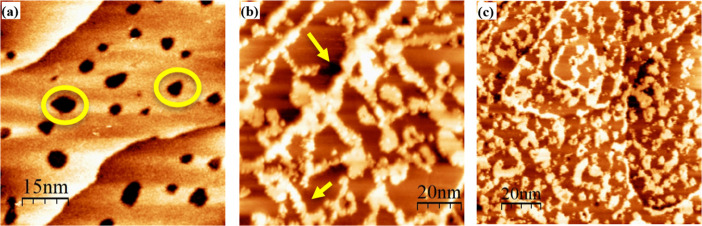
Representative STM images of DDT/TPT monolayers. Surfaces were rinsed with ethanol and dried under a nitrogen stream before imaging. Panels (a,b) show a binary SAM formed from immersing the sample in a 1 mM DDT solution at 78 °C followed by immersion in 0.1 mM ethanolic TPT solutions at 50 °C for 2 h (DDT(78)/TPT). In panel (a), a surface structure consistent with single-component DDT SAMs is observed, i.e. large flat terraces peppered with etch pits (circled), and in panel (b) we observe flat domains surrounded by clumpy, bright domains indicated by the arrow(s). Panel (c) shows a binary SAM formed from immersing the sample in a 1 mM DDT solution at room temperature followed by immersion in 0.1 mM ethanolic TPT solutions at 50 °C for 2 h (DDT­(RT)/TPT). It is challenging to ascertain height differences due to color saturation, but measurements ranged from 4–5 Å.

The second type of surface structure observed is shown in panels (b) and (c) of Figure 4. Here, we observe flat domains surrounded by brighter domains. The differing tunneling conditions between the two domains made it impossible to molecularly resolve both domains simultaneously. A similar monolayer structure was observed by Lussem et al.^86^ The results we obtained in our study did not exhibit the same high level of order as those reported in the Lussem study, most likely due to the fact that their study was conducted in ultrahigh vacuum conditions, which allowed for higher temperatures to be used to anneal the binary SAM structure. On the other hand, our experiments are carried out in ambient conditions, thus restricting the temperatures at which our SAMs could be annealed. We presume that the brighter domains are composed of either only TPT molecules, or a mixture of TPT and DDT. Control experiments were carried out in which single-component DDT monolayers were immersed in neat solvent for 2 h at 50 °C to mimic the deposition conditions of TPT (see Figure S2). As no changes to the DDT monolayer were observed with the control conditions, we attribute the change in the DDT monolayer to the adsorption of TPT. By far, this second type of surface structure was the one most commonly observed in DDT/TPT images.

#### TPT/DDT

Figures 5 and 6 show how, upon adsorption of DDT, we observed domains marked by features characteristic of either single-component TPT SAMs or single-component DDT SAMs based on the deposition temperature of DDT used. Unlike samples of DDT/TPT, we do not observe any regions where domains of the two compounds possibly coexist. Figure 5 shows representative STM images of binary TPT/DDT SAMs formed by immersing single-component TPT SAMs in DDT solutions at room temperature. These surfaces retained features of single-component TPT SAMs. Figure 5 shows a wide-field image of a sample in panel (a), and a zoomed-in domain structure panel (b). Panel (a) shows mainly a large terrace adjacent to several small terraces. Typical domain boundaries of TPT SAMs that are somewhat small recessions in the surface cover all the terraces, along with several gold adatom islands.

**Figure 5:**
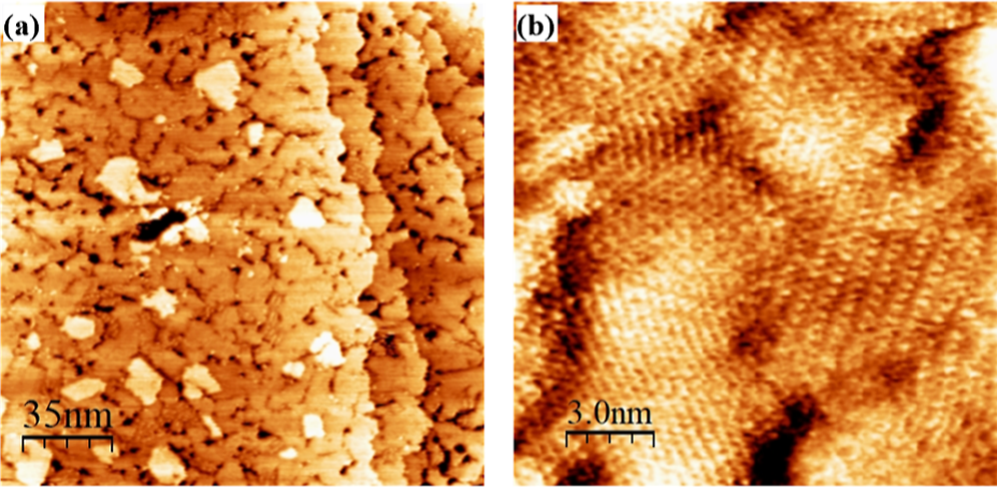
Representative STM images of binary TPT/DDT­(RT) SAMs in which single-component TPT features are observed. Panel (b) is a zoom in of panel (a) displaying the molecular resolution of a multiphase domain. Images are consistent with previously reported surface structures of TPT. These samples were prepared by immersing a flame-annealed gold surface in a 0.1 mM ethanolic solution of TPT at 50 °C for 2 h. Samples were then rinsed with ethanol and dried under a nitrogen stream before being immersed in a 1 mM ethanolic DDT solution for 1 h at room temperature. Prior to imaging, the sample was rinsed with ethanol and dried under a nitrogen stream.

**Figure 6:**
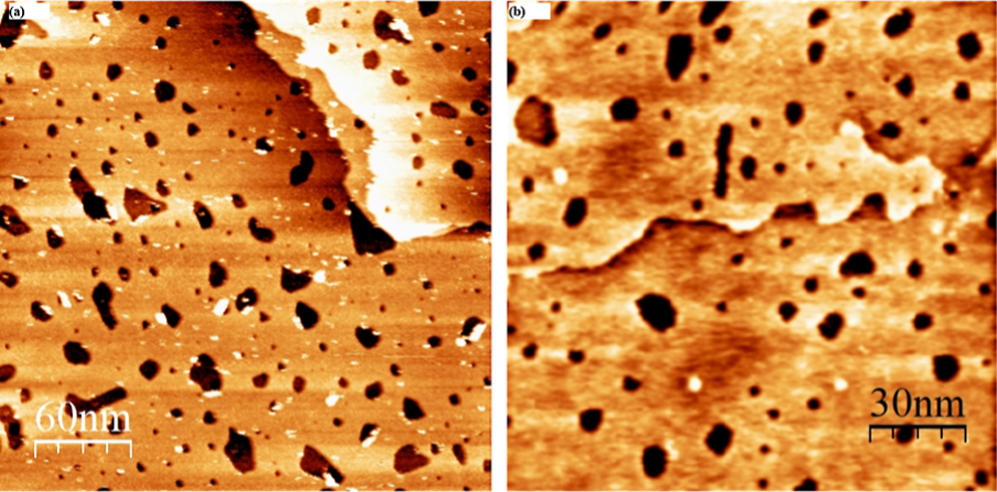
Panels (a) and (b) show representative STM images of binary TPT/DDT(78) monolayers displaying the displacement of TPT by DDT. These samples were prepared by immersing a flame-annealed gold surface in a 0.1 mM ethanolic solution of TPT at 50 °C for 2 h. The sample was then rinsed with ethanol and dried under a nitrogen stream before being immersed in a 1 mM ethanolic DDT solution for 1 h at 78 °C. Prior to imaging, the samples were rinsed with ethanol and dried under a nitrogen stream.

Samples in which the DDT solution was held at 78 °C resulted in the replacement of TPT by DDT. The resulting observed surface structures mirrored single-component DDT monolayers, though molecular resolution was not attained. Representative images of these surfaces are shown in Figure 6. In both panels, wide-scale areas depict multiple terraces that are peppered with etch pits. These results are consistent with previous studies in our group in which alkanethiols replace aryl thiols when the alkanethiol is deposited at an elevated temperature.^39,40^ Control experiments in which TPT SAMs were immersed in neat ethanol solvent for 1 h did not result in desorption or degradation of the monolayer (see Figure S3). Rather, control samples demonstrated surface structures consistent with single-component TPT SAMs. Thus, we conclude that the observed changes are due to the displacement of TPT rather than the exposure to an elevated temperature. There are several potential reasons as to why TPT is not displaced by DDT at lower temperatures. First, the aromaticity of TPT allows these compounds to engage in pi–pi stacking interactions with neighboring molecules.^87^ These interactions significantly enhance the cohesive forces within the SAM, making the monolayer more resistant to displacement than linear alkanethiols, which rely mainly on weaker van der Waals forces. Second, TPT molecules have a larger excluded volume than DDT molecules. This increased volume creates steric hindrance, physically blocking access of incoming DDT molecules to the gold surface. At lower temperatures, where molecular motion is more limited, this hindrance is even more effective. Finally, at lower temperatures, molecular mobility is reduced in general. In the case of rigid aromatic systems like TPT, surface diffusion and exchange processes are already sluggish due to their stiffness and strong intermolecular interactions. DDT molecules therefore cannot easily penetrate the SAM or displace bound TPT molecules.^88^

### XP Spectra

#### XPS of Single-Component DDT and TPT SAMs

Elemental analysis of single-component and binary SAMs was conducted via XPS. In Figure 7a, the XP spectrum of single-component, solution-deposited DDT(78) SAMs, shows a single S 2p_3/2_/S 2p_1/2_ doublet peak centered at 162 eV. This peak is indicative of a sulfur–gold bond.^16^ There is no signal present in the region of 165–167 eV which would indicate the presence of physisorbed organosulfur compounds on gold.^89–92^ In addition, the lack of peaks in the vicinity of 168 eV demonstrates the absence of oxidized sulfonate species.^74^

**Figure 7:**
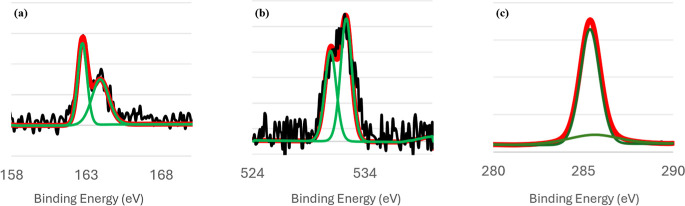
XP spectra of single-component DDT SAMs. The S 2p, O 1s, and C 1s signals are shown in panels (a), (b), and (c), respectively. On the graphs, the raw data signal is shown in black, the curves used to fit the data are shown in green, and the final fit is shown in red.

However, when investigating the presence of oxygen in the SAM, a sizable signal was discovered as shown in Figure 7b. A previous study by Willey et al.^90^ demonstrated DDT SAMs on Au(111) surfaces are prone to oxidation when samples are exposed to air and to light prior to being investigated with XPS. In that study, though, samples that were oxidized via air and light exposure displayed both oxidized sulfur peaks and oxygen peaks. This stands in opposition to our samples, which showed oxygen peaks but not oxidized sulfur peaks. Possible explanations for our observations include: a low concentration of oxidized sulfur species below the detection limit of the instrument, or the presence of surface-adsorbed oxygen. Previous reports from our research group do not show such prominent oxygen peaks associated with alkanethiolate monolayers, and we conclude that it is not an effect of our sample preparation methods or data collection techniques. Instead, we attribute this effect to the presence of disordered domains within DDT monolayers that allows for the adsorption of surface oxygen species to occur. To test this hypothesis, we have collected XPS for DDT SAMs prepared on both unannealed gold and for short deposition times to create an undersaturated SAM. Both molecular layers showed increased O/Au ratios compared to the DDT(78) samples prepared on annealed gold. The average O/Au ratio for the DDT(78) SAMs was 0.013, while the O/Au ratio for DDT(78) SAMs on unannealed gold was 0.019 and the O/Au ratio for DDT(78) deposited for 10 min to produce undersaturated layers was 0.014. Example data is included in Figure S4. The increased O/Au ratios and absence of oxidized sulfur or carbon species in the resulting spectra is consistent with the adsorption of surface oxygen due to disordered domains.

Lastly, the C 1s XP spectra of the DDT SAMs is shown in Figure 7c. The main contribution to the signal is positioned at 285.4 eV. Studies by Aagaard et al.,^93^ Willey et al.,^94^ and Fuxen et al.^95^ report the position of this peak for unadulterated DDT SAMs at just below 285 eV. In general, C 1s positions at signals lower than 285 eV indicate low-coverage monolayers.^81,96,97^ Though not identical, the position of our C 1s peak is in good agreement with values previously reported.

The S 2p signal of a single-component TPT SAM is displayed in Figure 8a. This XP spectrum shows a single S 2p_3/2_/S 2p_1/2_ doublet peak centered at 162 eV, which is indicative of a sulfur–gold bond. The shape and position of this peak are consistent with previous reports for this compound.^98,99^ We do not measure a large presence of physisorbed sulfur species nor oxidized sulfonate species in the 165–168 eV region, though there is a slight signal above the baseline in that region indicating the possible existence of these species on the surface.

**Figure 8:**
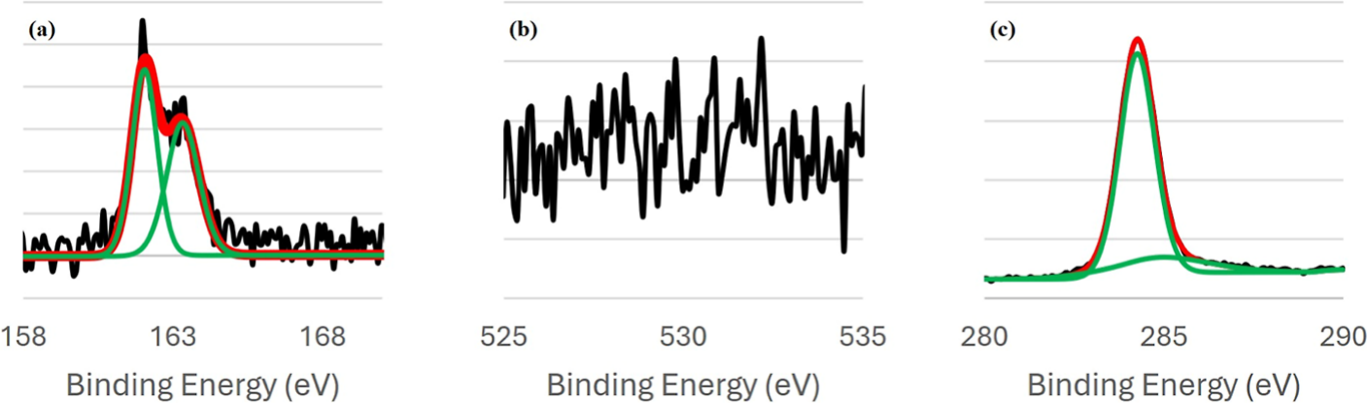
XP spectra of single-component TPT SAMs. The S 2p, O 1s, and C 1s signals are shown in panels (a), (b), and (c), respectively. On the graphs, the raw data signal is shown in black, the curves used to fit the data are shown in green, and the final fit is shown in red.

Investigation of the O 1s peak reveals a very slight baseline signal centered at 532 eV. The presence of this miniscule signal can also be attributed to the presence of surface adsorbed oxygen species, especially as our experiments are conducted in air. The absence of oxidized sulfonate species as well as oxygen species characterizes a robust TPT SAM that is not susceptible to oxidation, perhaps due to its surface ordering.

The C 1s XP spectra of the single-component monolayers are shown in Figure 8c. The main C 1s peak for the TPT monolayers is centered at 284.28 eV. This value is in excellent agreement with values previously reported by Fuxen et al.^95^ Additionally, other aryl thiol SAMs previously studied in our group^38–40^ and by others^100,101^ report the position C 1s peaks in this region.

#### XPS of Binary DDT and TPT SAMs

Figure 9 organizes the S 2p, O 1s, and C 1s peaks for the binary monolayer systems. XPS of binary SAMs was carried out using the 78 °C deposition temperature of DDT. Binary monolayers produced spectra with highly similar binding energies for the S 2p and C 1s peaks. The position of the S 2p peak was 162.08 eV for DDT/TPT SAMs, and 162.18 eV for TPT/DDT SAMs. The C 1s peak was identical for both systems, being centered just below 285 eV, at 284.98 eV. Though previous studies have reported shifts in the position of the C 1s peak between SAMs composed of alkanethiols and arylthiols, there is a large variability to this effect, and reported shifts tend to be minor.^38,71,81,102^ The only difference in the XP spectra of the binary SAMs was the presence or absence of a minor oxygen peak. DDT/TPT SAMs have a small baseline signal in the 532 eV region where O 1s peaks are usually centered, while TPT/DDT SAMs did not have any O 1s signal. It is expected that the XP spectra of binary SAMs are so similar, as the spectra of single-component SAMs are strongly correlated as well. The presence/absence of the O 1s peak between the single-component DDT and the binary SAMs can be attributed to an increase in adsorbates in binary SAMs that inhibits the adsorption of surface oxygen species.

**Figure 9:**
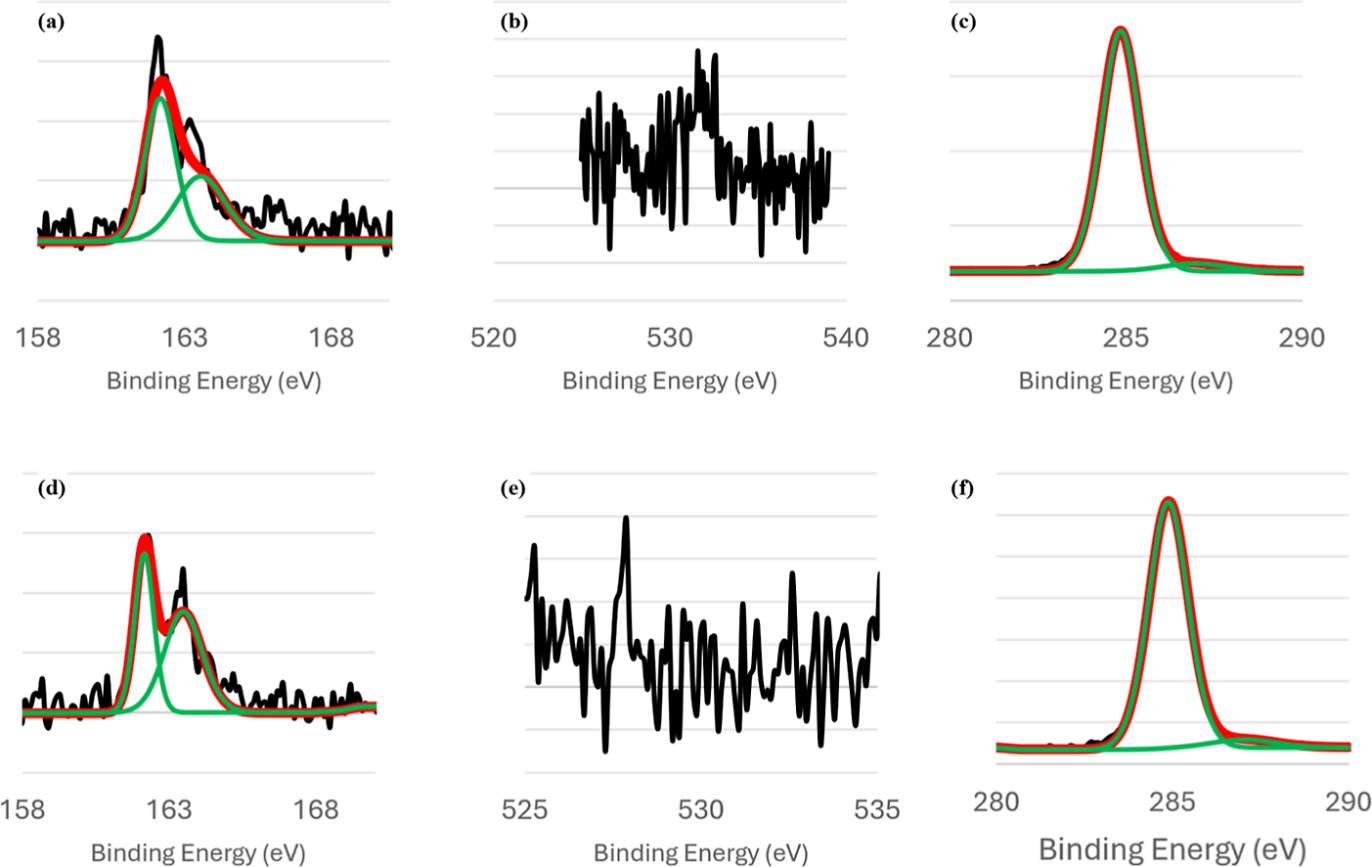
XP spectra of binary DDT and TPT SAMs. The S 2p, O 1s, and C 1s signals of DDT(78)/TPT are shown in panels (a–c), respectively. The same signals for TPT/DDT(78) are shown in panels (d–f). On the graphs, the raw data signal is shown in black, the curves used to fit the data are shown in green, and the final fit is shown in red.

#### Cyclic Votammetry of Single-Component and Binary SAMs

We performed reductive desorption to study the relative binding energies and levels of molecular layer homogeneity in the single component and mixed SAMs. Representative negative traces for each sample are shown in Figure 10 with peak quantification in Table 1. Reductive desorption of the DDT SAMs shows one primary sharp peak at −1.084 ± 0.014 V vs Ag/AgCl, labeled peak II for comparison between peaks of similar desorption potential. This peak potential is consistent with the desorption potential previously reported for SAMs formed from linear alkanethiols on Au(111) measured in basic conditions.^56,60,103^ In addition to the primary peak, smaller broad peaks around −1.18 and −0.80 V are present, labeled peaks I and III, respectively. The desorption potential can depend on several factors including the crystallographic orientation of the gold surface and the absence or presence of defect sites, and intermolecular forces within the SAM. Reductive desorption peaks at more negative potentials have been previously observed for SAMs on gold single crystals presenting less thermodynamically stable crystallographic surfaces compared to attachments on Au(111) surfaces so the peak at −1.18 V may be due to molecular components bound at defect sites.^57,58,104^ The current density appearing at a less negative potential is likely due to less well-ordered portions of the molecular layer in which weaker intermolecular forces are present. This may be due to the tendency of the molecular layers to allow for the adsorption of surface oxygen species, as described in the XPS section. The fwhm of the peak II at −1.084 V was 38 ± 9 mV, indicating a high degree of uniformity in the electrochemical environment of this domain. Prior work has shown that the peak width for reductive desorption of linear aliphatic alkanethiol SAMs on Au(111) will approach 20 mV for longer alkanethiols.^56^ The smaller broad peaks I and III have high levels of uncertainty in their widths due to the difficulty of establishing a consistent baseline, and indicate high levels of molecular layer inhomogeneity consistent with molecules that are less well ordered or bound at defect sites. Overall, these results support the STM results showing highly ordered domains of DDT as well as domain boundaries and areas of disorder.

**Figure 10:**
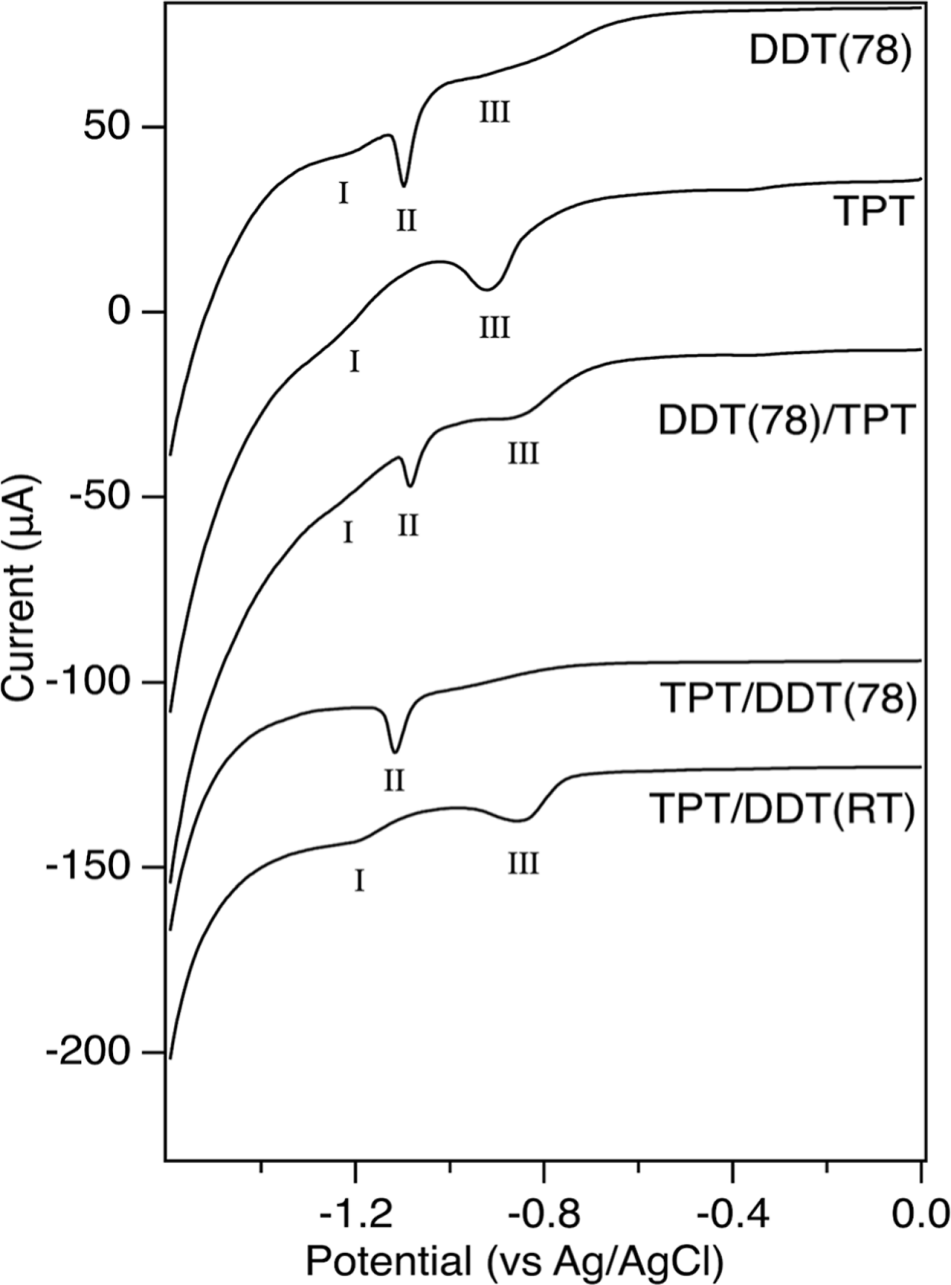
Reductive desorption voltammograms collected in 0.50 M KOH at 100 mV/s for each of the thiol-functionalized SAMs. Traces are offset on the *y*-axis for clarity. The peak labels I, II, and III are used to group peaks by similar desorption potential.

**Table 1:** Reductive Desorption Peak Quantification. Peak Positions are V vs Ag/AgCl^a^

SAM	peak I position (V) Width (mV)	peak II position (V) width (mV)	peak III position (V) Width (mV)
DDT(78)	–1.18 ± 0.04, 69 ± 35	–1.084 ± 0.014, 38 ± 9	–0.80 ± 0.09, 190 ± 40
TPT	–1.258 ± 0.012, 110 ± 30		–0.91 ± 0.04, 117 ± 17
DDT(78)/TPT	–1.203 ± 0.010, 80 ± 20	–1.078 ± 0.005, 33 ± 3	–0.85 ± 0.02, 167 ± 9
TPT/DDT(78)		–1.114 ± 0.008, 38 ± 9	
TPT/DDT(RT)	–1.21 ± 0.02, 110 ± 10		–0.88 ± 0.03, 100 ± 20

a Peaks are placed in columns of similar desorption potential for comparison.

Reductive desorption measurements of the single component TPT SAMs show a primary peak III at −0.91 ± 0.04 V, with a smaller broad shoulder peak I at −1.258 ± 0.012 V. The position of the primary peak III is at a significantly less negative potential than the pure DDT SAM primary peak, indicating a less stable molecular attachment to the gold surface. These findings are consistent with results showing that aryl thiols exhibit less negative desorption potentials than aliphatic thiols because their electron withdrawing ability favors the acceptance of electrons from the gold surface that occurs during reductive desorption.^21^ The fwhm of peak III is 117 ± 17, indicating a significant decrease in the homogeneity of the electrochemical environment for these molecules compared to the pure DDT molecular layer. The heterogeneity of electrochemical environments observed through the width of the primary TPT peak III is consistent with the observation of multiple TPT domain structures in the STM images. The small, broad, peak I at 1.258 ± 0.012 V indicates the presence of molecules in substantially more stable binding environments. Similar to the DDT reductive desorption data, this peak I is likely due to molecules attaching at less thermodynamically stable sites on the gold surface.^57,105,106^ While TPT reductive desorption does not appear in the literature, the −0.91 V peak III potential measured in this work is close to the −0.926 V desorption potential measured for biphenylthiol. Biphenylthiol also exhibited a relatively broad desorption peak that was attributed to poor molecular packing.^107^

The DDT(78)/TPT SAM reductive desorption measurements show a sharp peak II at −1.078 ± 0.005 V, and a second significant peak III at −0.85 ± 0.02 V. We also observe a very small, broad peak I at −1.203 ± 0.010 V that was quantifiable on four of the five measured samples. The presence of the substantial peak II at −1.078 V and peak III −0.85 V indicate the presence of at least two domains for the SAM. Comparison of the location of the peaks with the single component molecular layer desorption potentials suggests that the peak II at −1.078 V can be attributed to DDT, while the peak III at −0.85 V is at a potential similar to, but slightly less negative than the pure TPT molecular SAM. However, the DDT samples also have observed current density at −0.80 V so the peak III at −0.85 V cannot be solely attributed to TPT. The peak III at −0.85 V also has a significantly higher fwhm than the single component TPT SAMs, indicating a high level of inhomogeneity in the electrochemical environment of the molecules, possibly due to the presence of both DDT and TPT at a similar desorption potential. The very small peak I at −1.203 V has a similar potential to the minor peaks observed in both the single component samples so we also cannot assign the peak to a specific molecule.

We can use the relative peak areas of the three different DDT(78)/TPT SAM reductive desorption peaks to further understand the contributions of these three molecular domains. The peak II at 1.078 ± 0.005 V, assigned to DDT, contributed 29 ± 8% of the total current density, while the peak III at −0.85 V, assigned to TPT or less well-ordered DDT, contributed 66 ± 4%. The small peak I at −1.203 V contributed 5 ± 4% of the current density. While it was not possible to achieve molecular resolution for domains of single component DDT using STM in these DDT/TPT mixed samples, the reductive desorption data supports the identification of well-ordered single component DDT domains. In addition to the DDT domains, reductive desorption indicates that the majority of the molecular layer is composed of domains of TPT, less well-ordered DDT, or a combination of both molecules, and a small population of molecules is bound in less thermodynamically stable sites of the gold surface. These results are consistent with the varied STM domains observed and allow us to more definitively identify the single component DDT presence in particular.

Reductive desorption measurements of room temperature DDT deposition followed by TPT deposition were also collected. The DDT­(RT)/TPT reductive desorption did not show significantly different peaks than the DDT(78)/TPT reductive desorption, which was consistent with the corresponding STM images, shown in Figure 4. A representative reductive desorption trace of DDT­(RT)/TPT is shown in Figure S5.

Reductive desorption of TPT/DDT mixed molecular layers was collected at DDT deposition temperatures of 78 °C and room temperature due to the differing STM results at each condition. The results collected for DDT deposition at 78 °C have a primary peak II at −1.114 ± 0.008 V. The peak potential and width are both consistent with a molecular layer of DDT. While the samples showed small, very broad current density at higher and lower potentials, the peaks were not reproducibly distinguishable from the baseline for quantification. This is consistent with the STM results showing replacement of TPT with DDT in the higher temperature DDT deposition.

We observe substantial differences in the TPT/DDT­(RT) reductive desorption compared to the higher temperature DDT deposition. The primary peak III is present at −0.88 ± 0.03 V, with a broad peak I at −1.21 ± 0.02. Both the position and widths of these peaks are consistent with the reductive desorption data collected for the single component SAMs of TPT. The presence of a significant peak III at −0.88 V and absence of a peak II around −1.1 V is consistent with the presence of TPT on the surface and an absence of DDT domains. This result matches the STM finding showing that DDT does not displace TPT molecular layers when deposited at room temperature.

Figure 11 shows cyclic voltammograms (CVs) collected for the single component and mixed SAMs of DDT and TPT in a ferricyanide electrolyte solution. A CV collected under the same conditions for a bare polycrystalline gold surface is included in Figure S6 to confirm the presence of reversible oxidation and reduction peaks on a bare gold surface. The plots of both the single complement and mixed SAMs show a capacitive shape without measurable oxidation or reduction peaks. The absence of significant peaks indicates that the redox couple is inhibited and indicates an absence of large defects in the molecular layer. The blocking behavior for DDT is consistent with prior studies.^3^ The slight “s” shape of the curves for the TPT and mixed SAMs indicates more moderate blocking behavior for these surfaces. These molecular layers do not have significantly different cathodic currents to indicate differences in blocking ability. Prior studies of ferricyanide CV for TPT and related aromatic SAMs have reported varied results including relatively little blocking for TPT^67^ and biphenyl thiols^68^ and substantial blocking for mono or biphenyl-based aromatic thiols.^108–110^ The substantial blocking observed in our studies may be due to the elevated temperature used during TPT deposition, compared to the room temperature deposition used in Rubinstein’s work, as our control CVs collected for TPT deposition at room temperature show significant anodic and cathodic current, shown in Figure S7.^67^

**Figure 11:**
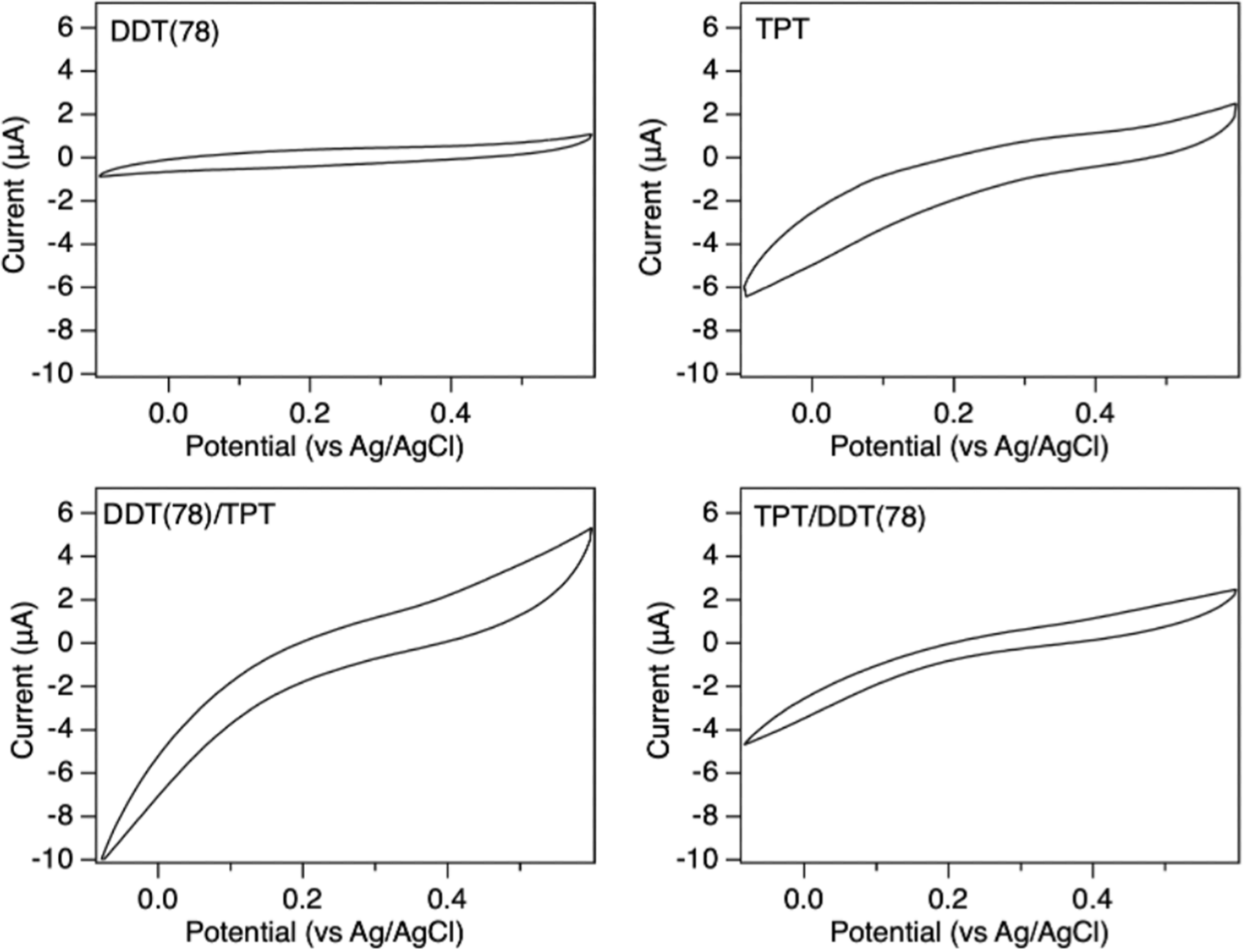
Cyclic voltammograms of thiol-functionalized Au(111) in 1 mM Fe­(CN)_6_^3–/4–^ and 1 M KCl collected at 100 mV/s. Each graph is plotted with the same *y*-axis scale to facilitate comparison.

## Conclusion

Binary SAMs of DDT and TPT on the (111) surface of gold were successfully characterized in ambient conditions. By using two different deposition temperatures of DDT and varying the sequence of deposition, four distinct binary monolayers were generated and investigated using STM, XPS, RD, and CV. The surface structures of binary SAMs reflected: maintaining the structure of the initial SAM, adsorption of the secondary compound at domain boundaries, or substitution of the initial SAM at higher deposition temperatures. XPS and RD analyses showed that binary SAMs had nearly identical elemental compositions, and distinct domains of binary SAMs that aligned with STM findings.

As SAM functionalities expanded and researchers explored exciting new frontiers, opportunities to address key questions about SAM behavior were left open for further investigation. At the heart of these unanswered questions is a fundamental gap in our understanding of two-dimensional nanoscale mixtures. However, from contaminants to naturally occurring blends, chemical compounds are often found intermingled with other compounds on surfaces. This necessitates an understanding of mixtures at the molecular level. Understanding the chemistry by which compounds mix on surfaces can provide unprecedented top-down control of two-dimensional nanoscale materials. We have approached the question of two-dimensional mixing by constructing a library of binary SAMs to identify the chemical interactions that guide the formation of surface structures. This study represents the fourth entry in our library of binary SAMs. We observe several similarities and differences between binary SAMs of alkanethiols and arylthiols, and continue to emphasize how deposition conditions play a critical role in determining the structural outcomes in a binary SAM. Future studies will continue to explore library entries, and seek a deeper understanding of the role of deposition variables of concentration and temperature.

## Supplementary Material



## Notes

Additional STM, XPS, and electrochemistry figures (PDF)
