# Supplementary material for: Binary Monolayers Formed from the Sequential Adsorption of Terphenylthiol and Dodecanethiol on Gold
Source: J Phys Chem C Nanomater Interfaces. 2025 May 19;129(21):9844–56. doi: 10.1021/acs.jpcc.5c00571 (PMC12128101; doi:10.1021/acs.jpcc.5c00571)
Supplement: Supplementary file 1 [file jp5c00571_si_001.pdf]

Binary Monolayers Formed from the Sequential Adsorption of Terphenylthiol and Dodecanethiol on Gold

Elizabeth Garrett, Sabrina Tang, Emma K. Canning, Daniel J. Williams, Aidan F. Bergin, Elaine Kelly, Luke Wadzinski, Emma R. Robinson, Alissandra Conlon, Jack Sette-Ducati, Sophia Renzi, Elizabeth C. Landis*, and L. Gaby Avila-Bront*

Department of Chemistry, College of the Holy Cross
1 College St., Worcester, MA, 01610

*To whom correspondence should be addressed.
E-mail addresses: lavila@holycross.edu, elandis@holycross.edu

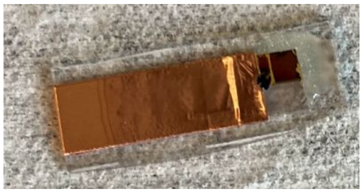

Figure S1. Picture of a Au(111) electrode mounted for electrochemical measurements with a copper tape top contact.

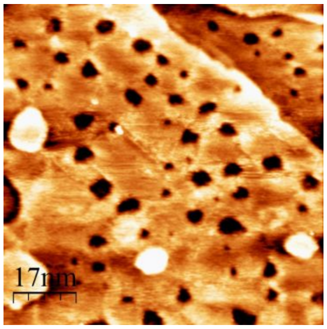

Figure S2. Representative STM image of a DDT SAM formed by immersing a flame-annealed gold substrate in a 1 mM ethanolic DDT solution for 1 h at room temperature. Following this deposition, the sample was then immersed in neat ethanol solvent for 2 h at 50 °C to mimic the deposition conditions of TPT monolayers. As these control experiments showed that the DDT SAM was not restructured to include additional adsorbates at domain boundaries, we attributed the presence of such features to be caused by the adsorption of TPT.

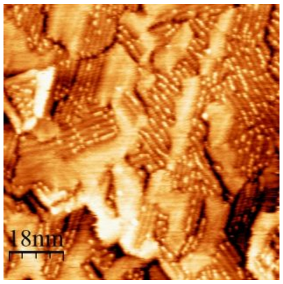

Figure S3. Representative STM image of a TPT SAM formed by immersing a flame-anneled gold substrate in a 0.1 mM ethanolic TPT solution for 2 h at 50 °C. Following this deposition, the sample was then immersed in neat ethanol solvent for 1 h at 78 °C to mimic the deposition conditions of DDT(78) monolayers. As these control experiments showed that the TPT SAM was not displaced, we attributed the displacement of TPT to be caused by the presence of DDT at a higher deposition temperature of 78 °C.

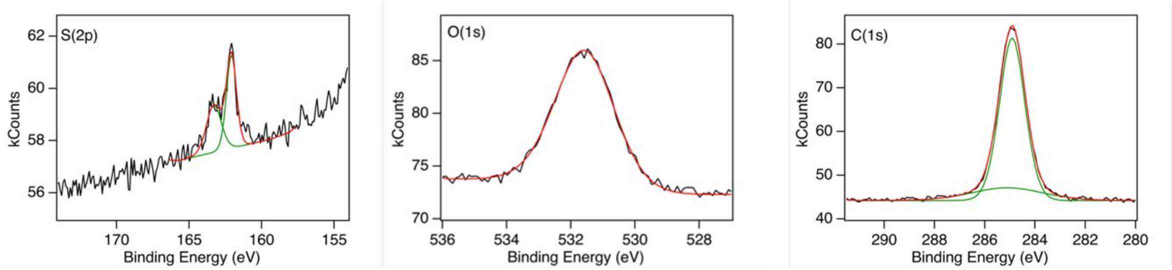

Figure S4. Representative XPS collected for DDT deposited for 10 minutes on annealed Au at 78 ℃ to form an undersaturated SAM. The increased oxygen level combined with the absence of oxidized carbon or sulfur support the hypothesis that surface-adsorbed oxygen is present in the DDT single component molecular layers.

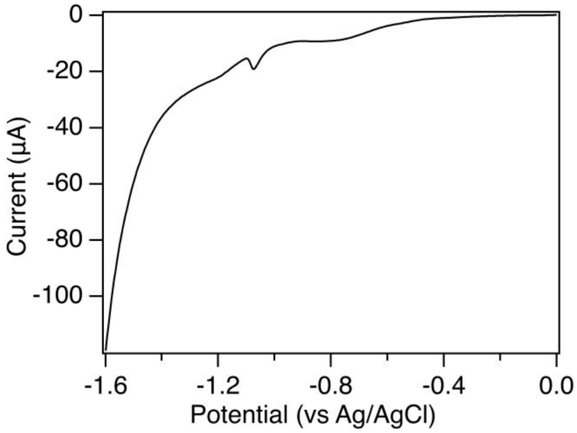

Figure S5. Reductive desorption voltammograms collected in 0.50 M KOH at 100 mV/s for a DDT(RT)/TPT molecular layer.

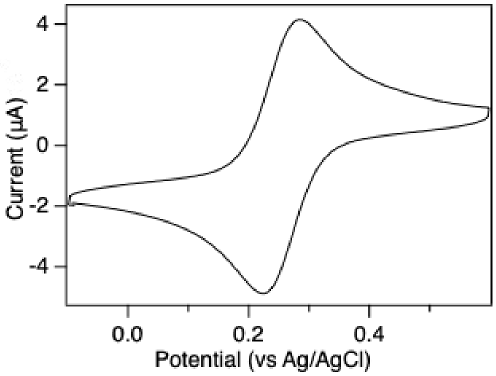

Figure S6. Cyclic voltammetry of a polycrystalline bare gold electrode in 1 mM $Fe(CN)_6^{3-/4-}$ and 1 M KCl collected at 100 mV/s to confirm the presence of oxidation and reduction peaks on the bare gold surface.

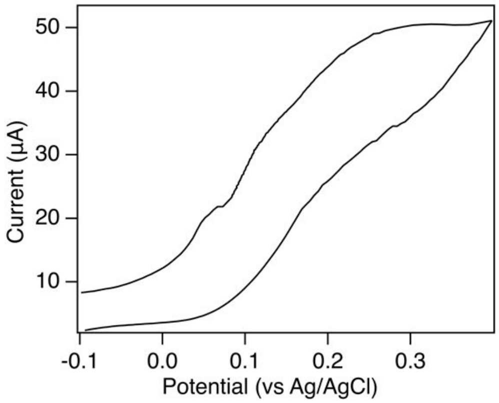

Figure S7. Cyclic voltammetry of TPT(RT) molecular layers in 1 mM $Fe(CN)_6^{3-/4-}$ and 1 M KCl collected at 100 mV/s. This data shows substantially higher anodic and cathodic peaks compared to the 78 °C TPT depositions shown in Figure 11.
